# The Beneficial Fungus *Mortierella hyalina* Modulates Amino Acid Homeostasis in Arabidopsis under Nitrogen Starvation

**DOI:** 10.3390/ijms242216128

**Published:** 2023-11-09

**Authors:** Nataliia Svietlova, Michael Reichelt, Liza Zhyr, Anindya Majumder, Sandra S. Scholz, Veit Grabe, Anne Krapp, Ralf Oelmüller, Axel Mithöfer

**Affiliations:** 1 Research Group Plant Defense Physiology, Max Planck Institute for Chemical Ecology, 07745 Jena, Germany; nsvietlova@ice.mpg.de (N.S.); yzhyr@ice.mpg.de (L.Z.); amajumder@bot.uni-kiel.de (A.M.); 2 Department of Biochemistry, Max Planck Institute for Chemical Ecology, 07745 Jena, Germany; reichelt@ice.mpg.de; 3 Department of Plant Physiology, Matthias-Schleiden-Institute, Friedrich-Schiller-University, 07743 Jena, Germany; s.scholz@uni-jena.de (S.S.S.); ralf.oelmueller@uni-jena.de (R.O.); 4 Microscopic Imaging Service Group, Max Planck Institute for Chemical Ecology, 07745 Jena, Germany; vgrabe@ice.mpg.de; 5 Institut Jean-Pierre Bourgin (IJPB), AgroParisTech, INRAE, Université Paris-Saclay, 78000 Versailles, France; anne.krapp@inrae.fr

**Keywords:** nitrate/nitrogen deficiency, nitrate transporters (NRTs), free amino acids, *Mortierella hyalina*, plant–fungus interactions, endophytic fungi

## Abstract

Non-mycorrhizal but beneficial fungi often mitigate (a)biotic stress-related traits in host plants. The underlying molecular mechanisms are mostly still unknown, as in the interaction between the endophytic growth-promoting soil fungus *Mortierella hyalina* and *Arabidopsis thaliana*. Here, abiotic stress in the form of nitrogen (N) deficiency was used to investigate the effects of the fungus on colonized plants. In particular, the hypothesis was investigated that fungal infection could influence N deficiency via an interaction with the high-affinity nitrate transporter NRT2.4, which is induced by N deficiency. For this purpose, Arabidopsis wild-type *nrt2.4* knock-out and NRT2.4 reporter lines were grown on media with different nitrate concentrations with or without *M. hyalina* colonization. We used chemical analysis methods to determine the amino acids and phytohormones. Experimental evidence suggests that the fungus does not modulate *NRT2.4* expression under N starvation. Instead, *M. hyalina* alleviates N starvation in other ways: The fungus supplies nitrogen (^15^N) to the N-starved plant. The presence of the fungus restores the plants’ amino acid homeostasis, which was out of balance due to N deficiency, and causes a strong accumulation of branched-chain amino acids. We conclude that the plant does not need to invest in defense and resources for growth are maintained, which in turn benefits the fungus, suggesting that this interaction should be considered a mutualistic symbiosis.

## 1. Introduction

The uptake of nitrogen via the roots is essential for plant growth. Nitrogen plays a special role in plant growth and productivity and is a crucial nutrient for plants, incorporated as the main building block of amino acids, proteins and many secondary metabolites. Plants efficiently acquire nitrogen and distribute it from source to sink organs under various environmental conditions [1,2]. It is usually absorbed in inorganic form, as ammonium or nitrate (NO_3_^−^). The latter is the most important source of nitrogen. While ammonium can be directly assimilated into glutamine in the root, nitrate is first transported to the shoot. Nitrate is then reduced into ammonium in various enzymatic steps, transferred to the amino acid glutamine using glutamine synthetase and further introduced into the metabolism by aminotransferases. From inorganic nitrate acquisition to organic nitrogen translocation and distribution, plants have evolved different strategies and systems [3,4,5,6,7,8,9,10,11]. An interesting aspect in this context is the initial uptake of nitrate, especially under nitrate deficiency. In order to survive in soil environments with different amounts of nitrate present, plants have evolved different transport systems to take up nitrate, which have been described in detail [3,4,5,6]. Briefly, two classes of transporter systems are involved in nitrate uptake: nitrate transport systems with high affinity, called High-Affinity Transporter Systems (HATS), and with low affinity called Low-Affinity Transporter Systems (LATS) [3,9,12]. So far, four nitrate transporter families have been identified, those are NRT1/PTR (NPF, nitrate transporter 1/peptide transporter family), NRT2 (nitrate transporter), CLC (chloride channels) and SLAC1/SLAH (slow anion channel-associated 1 homologs) [5,8]. Among these, only NRTs are involved in nitrate uptake from the soil, while NRT1 transporters are mainly LATS with different intracellular localizations. Most NRT2 transporters belong to HATS and are often localized in the plasma membrane [5]. The NRT2 family has a major contribution to the nitrate influx into roots. NRT2.1, NRT2.2, NRT2.4 and NRT2.5 are important for plants to survive with nitrate limitation. Here, NRT2.1 and NRT2.4 play a major role in the maintenance of optimal plant growth under different nitrate conditions. While NRT2.1 is a main component of HATS both under low nitrate conditions and with a nitrate supply, expression of *NRT2.4* was observed only in lateral roots and younger parts of the main root under nitrogen starvation. This revealed that NRT2.4 is specifically involved in the response to nitrate starvation [12].

Of course, such nitrate or nitrogen deficiencies must be recognized and managed by the plant [13]. The ability to monitor the cellular N status is essential for maintaining metabolic homeostasis, growth and development in plants. Candidates that are considered for the role of N sensory systems and further signaling to appropriate physiological responses include the target of rapamycin (TOR) signaling pathway, the general control non-derepressible 2 (GCN2) pathway, the family of glutamate-like receptors (GLRs) and the plastidic P_II_-dependent pathway [14]. All these putative candidates have in common a hypothesized role in binding amino acids. Strikingly, the widely distributed P_II_ is non-functional in Brassicaceae, including Arabidopsis [15]. However, despite recent progress in understanding the function and in part the mode of action of these signaling systems, there is still lacking knowledge concerning to what extent they contribute to the process of N status monitoring in plants [14].

It is already known that a number of beneficial microorganisms, in particular endophytic fungi, can positively influence the growth of many plants under stress. Endophytic fungi are facultative symbionts of plants. Depending on the particular host plant, developmental stage, nutrition and other environmental factors, they may interact with their host as mutualistic symbionts, as commensals or as latent pathogens [16]. Unlike mycorrhizal fungi, their growth is not synchronized with the development of their hosts [17]. Plants colonized by endophytic fungi often show improved growth, better productivity and induced resistance against biotic attackers [18,19,20,21,22,23]. For example, co-cultivation with beneficial fungi such as *Mortierella hyalina* can promote the growth of *Arabidopsis thaliana* [24,25]. *M. hyalina* is an endophytic fungus belonging to the order *Mortierellales*, the largest genus within *Mucoromycota* [26]. Dominant fungal communities in natural ecosystems harbor various members of the order *Mortierellales* [27], including the non-pathogenic genus *Mortierella.* Species of *Mortierella* live as saprotrophs in soil, on decaying leaves and other organic material. In addition, many of those colonize roots of a wide variety of plant species and stimulate growth and biomass production in the aerial parts of plants [23]. However, very often, the underlying molecular mechanisms are still unknown.

The positive influence of beneficial fungi on stressed plants is a well-known phenomenon. This also applies to the positive effect of beneficial fungi on plants under N starvation. Although the role of nitrate transporters in nitrate deficiency has been demonstrated [28], a possible influence of beneficial fungi on such NRTs has not yet been shown. Therefore, the aim of this study was to investigate and understand the putative role of a beneficial fungus, *M. hyalina*, on nitrate uptake by a high-affinity nitrate transporter such as NRT2.4 and furthermore on nitrogen metabolism, exemplified by amino acid metabolism, in colonized Arabidopsis plants under N starvation. It is shown here for the first time that the fungus does not affect the expression of NRT.2.4. Instead, *M. hyalina* can supply nitrogen to N-starved plants. Furthermore, we show that the presence of *M. hyalina* can restore the amino acid homeostasis disturbed by nitrogen deficiency in both the shoots and roots of the host plant.

## 2. Results

### 2.1. Effect of Mortierella hyalina Colonization on Fresh Weight of Arabidopsis Plants under Nitrogen Starvation

**Figure 1:**
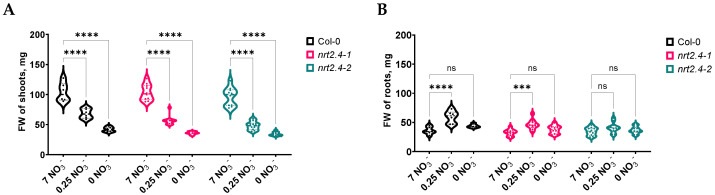
Fresh weight of shoots (**A**) and roots (**B**) of *Arabidopsis thaliana* WT and ko mutant plants during NO_3_^−^ starvation. Two-week-old seedlings pre-grown on full NO_3_^−^ (7 mM NO_3_^−^) medium were further incubated on a different NO_3_^−^ medium (N-free, 0 mM NO_3_^−^; N-low, 0.25 mM NO_3_^−^; N-complete, 7 mM NO_3_^−^) for another 10 d. Each replicate represents a sum of 3 seedlings. Two-way ANOVA with Dunnett’s multiple comparison test; n = 6–10; *** *p* < 0.001; **** *p* < 0.0001; ns: not significant.

A first study of the root growth features in the different Arabidopsis lines showed that even under a high NO_3_^−^ concentration (7 mM), the total length of the main and lateral roots was reduced on the 6th and even more on the 10th day of incubation in the *NRT2.4* knock-out (ko) mutant lines (*nrt2.4-1* and *nrt2.4-2*) compared to the wild type (WT, Col-0). The fresh weight (FW) of the shoots and roots in all these lines was detected to be more dependent on the nitrate concentration than on the genotype (Figure 1).

**Figure 2:**
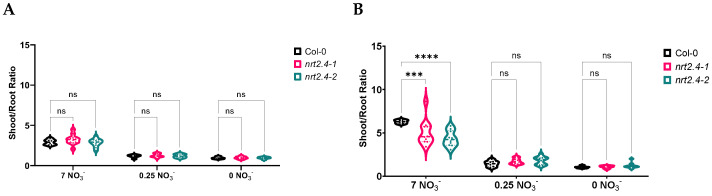
Shoot/root ratio of *Arabidopsis thaliana* WT and ko mutant plants without (**A**) or with (**B**) *Mortierella hyalina* co-cultivation during NO_3_^−^ starvation. Two-week-old seedlings pre-grown on full NO_3_^−^ (7 mM NO_3_^−^) medium were further incubated on a different NO_3_^−^ medium (N-free, 0 mM NO_3_^−^; N-low, 0.25 mM NO_3_^−^; N-complete, 7 mM NO_3_^−^) and were not (**A**) or were (**B**) co-cultivated with *M. hyalina* for another 10 d. Each replicate represents the sum of three seedlings. Two-way ANOVA with Dunnett’s multiple comparison test; n = 6–10; *** *p* < 0.001; **** *p* < 0.0001; ns: not significant.

In the WT and mutant lines, 10 d of nitrate deficiency reduced the FW in the shoots, and the lower the nitrate concentration, the lower the weight (Figure 1A). In contrast, the roots’ FW was not negatively affected (Figure 1B). Upon infection with *M. hyalina*, the shoots and roots showed the same trend as the non-infected plants, but with a clearly lower FW. These results suggest that the fungus somehow competes with the plant for nitrogen, resulting in a lower FW for the plant. The reduction in root FW was most pronounced on complete nitrate media, which is reflected in the shoot/root ratio (Figure 2). There was no difference in shoot/root ratio between the non-infected ko mutants and WT plants, only a slight reduction due to the nitrate concentrations (Figure 2A). However, upon *M. hyalina* colonization, on complete nitrate media, the WT plants showed a significantly higher shoot/root ratio compared with the mutant lines (Figure 2B). However, the shoot/root ratio in the mutant lines grown with *M. hyalina* is almost on the same level as in the non-infected control plants.

### 2.2. Effect of Mortierella hyalina Colonization on Phytohormones in Arabidopsis Plants under Nitrogen Starvation

**Figure 3:**
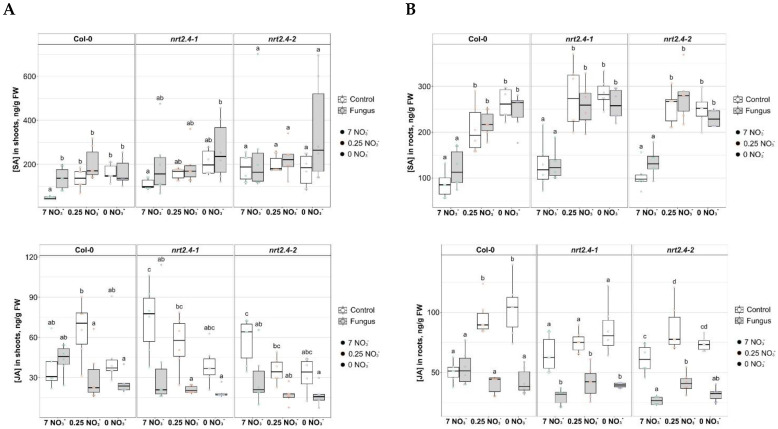

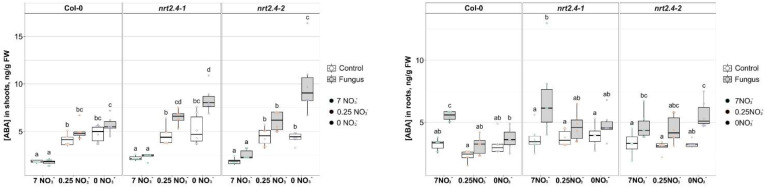
Phytohormone contents in shoots (**A**) and roots (**B**) of *Arabidopsis thaliana* WT and ko mutant plants with and without *M. hyalina* co-cultivation during NO_3_^−^ starvation. Two-week-old seedlings pre-grown on full NO_3_^−^ (7 mM NO_3_^−^) medium were further incubated on different NO_3_^−^ medium (N-free, 0 mM NO_3_^−^; N-low, 0.25 mM NO_3_^−^; N-complete, 7 mM NO_3_^−^) and co-cultivated without and with *M. hyalina* for another 10 d. The line from the box’s ends extends from the first and the third quartile, the line in the middle represents median. Two-way ANOVA with Tukey’s post hoc test, n = 5–6 (data were transformed when needed). Different letters indicate significant differences (*p* < 0.05) across groups.

*M. hyalina* has been described as a beneficial fungus. In principle, however, the plant could also recognize the infection with *M. hyalina* as an attack. To investigate this possibility, defense and stress-related phytohormones (salicylic acid, SA; jasmonate, JA; abscisic acid, ABA) were analyzed (Figure 3). It became clear that in all Arabidopsis genotypes, the roots (Figure 3B) and not shoots (Figure 3A) showed a significant increase in SA under nitrate deficiency stress, but not under *M. hyalina* infection. JA was also found to have an effect on the phytohormone content. A higher JA content was found in the roots and shoots of WT plants under nitrate deficiency; this effect was attenuated by infection with *M. hyalina*. In both *nrt2.4* mutant lines, the JA-reducing effect of the fungus was also visible (Figure 3). ABA was also weakly but significantly induced in the shoots but not in the roots when nitrate was deficient. The presence of *M. hyalina* slightly increased the ABA levels in all cases (Figure 3). These results clearly show that the plant neither recognized *M. hyalina* as a pathogen nor activated any defense mechanism against the fungus.

### 2.3. Mortierella hyalina Colonization Can Provide Nitrogen to Arabidopsis Plants under Nitrogen Starvation

Next, we investigated whether and to what extent the fungus may provide nitrogen to the plant. Compared with the incubation with the unlabeled fungus, much higher ^15^N levels were detected in the Arabidopsis shoots (Table 1). The lower the nitrate concentration in the medium, the higher the uptake of fungus-provided ^15^N. A ^15^N level up to 9895 times higher was found in shoots growing without nitrate, 829 times higher with 0.25 mM nitrate and 52 times higher with 7 mM nitrate. This clearly indicted a transport of ^15^N from the fungal hyphae to the plant roots and from the roots to the shoots. This effect is much more intense if no NO_3_^−^ is available in the medium, suggesting an exchange of nutrients only when it is necessary.

### 2.4. Effect of Nitrogen Starvation on NTR2.4 Gene Induction with/without Mortierella hyalina Colonization

**Figure 4:**
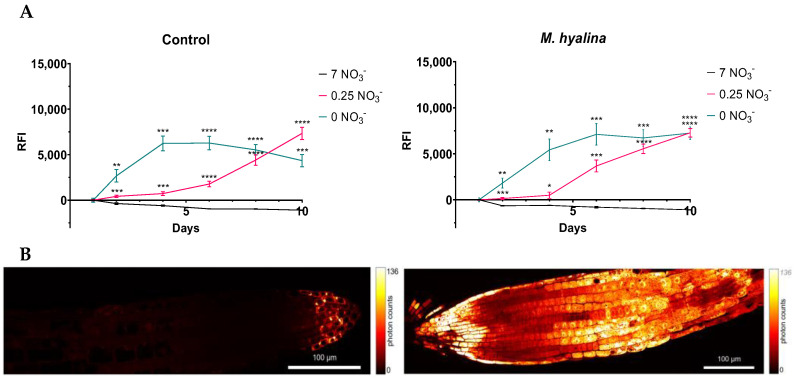
Relative fluorescence intensity (RFI) (**A**) and confocal microscopy images (**B**) of roots of *Arabidopsis thaliana ProNRT2.4:GFP* transgenic plants. (**A**) Two-week-old seedlings pre-grown on full NO_3_^−^ (7 mM NO_3_^−^) medium were further incubated on different NO_3_^−^ media (N-free (0 mM NO_3_^−^), N-low (0.25 mM NO_3_^−^) and N-complete (7 mM NO_3_^−^)) and were or were not co-cultivated with *M. hyalina* for the indicated time. Two-way ANOVA with Dunnett’s multiple comparison test; n = 8. *M. hyalina*: Mixed-effects model (REML) with Dunnett’s multiple comparison test; n = 4–8; the error bars indicate standard error (SE); * *p* < 0.05; ** *p* < 0.01; *** *p* < 0.001; **** *p* < 0.0001; ns: not significant. (**B**) Two-week-old seedlings pre-grown on full NO_3_^−^ (7 mM NO_3_^−^) medium were further incubated on N-complete (7 mM NO_3_^−^, left) or N-free (0 mM NO_3_^−^, right) for 2 d. STEDYCON microscopy and STEDYCON processing software (exc. laser 488 nm, detector 526 nm). To compare the fluorescence intensity in these variants, the raw images were aligned by photon count to the same level in the two images.

In order to find out whether *M. hyalina* somehow affects the high-affinity nitrate uptake system (HATS), the *ProNRT2.4:GFP* reporter line was employed (Figure 4). While fluorescence was detectable rapidly upon transfer in seedlings grown without nitrate with a peak after 4–6 d, with 7 mM nitrate in the medium, no induction occurred. Strikingly, the presence of *M. hyalina* had no obvious effect on the N-deficiency-induced expression of *NRT2.4*. This suggests that *M. hyalina* does not influence NRT2.4-mediated nitrate uptake.

Additional qPCR experiments supported the *NRT2.4* gene induction over time under nitrate deficiency in the Arabidopsis Col-0 roots (Figure S2A). A 12.2-fold increase of *NRT2.4* transcripts was detected after 2 d on the media without nitrate and still a 3.4-fold increase on 0.25 mM nitrate, all compared to the controls grown on 7 mM nitrate. After 10 d, *NRT2.4* gene induction did not increase anymore and even decreased without nitrate, suggesting an early but transient induction of this transporter (Figure S2A). Strikingly, an interesting finding was that the expression of the high-affinity nitrate transporter *NRT2.5* under nitrate starvation was regulated differently. In contrast to *NRT2.4*, the *NRT2.5* expression was lower at d 2 than at d 10 (from 66.7- to 255-fold, respectively) on the media with and without nitrate (Figure S2B).

### 2.5. Effect of Mortierella hyalina Colonization on Amino Acid Pools in Arabidopsis Plants under Nitrogen Starvation

**Figure 5:**
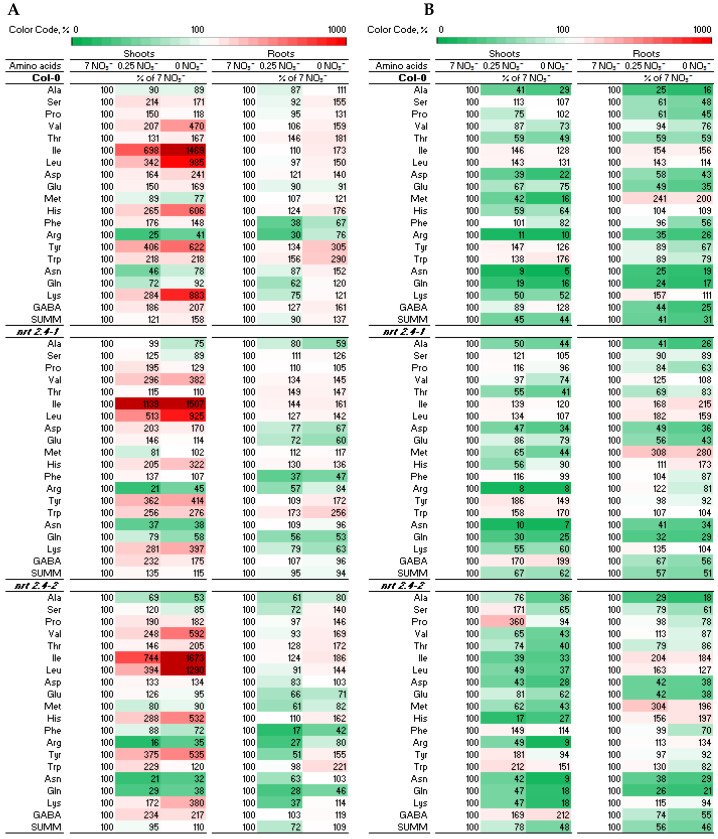
Heat map of amino acid level in shoots and roots in *A. thaliana* WT and ko mutants co-cultivated without (**A**) or with (**B**) *Mortierella hyalina* during NO_3_^−^ starvation. Two-week-old seedlings pre-grown on full NO_3_^−^ (7 mM NO_3_^−^) medium were further incubated on different NO_3_^−^ media (N-free, 0 mM NO_3_^−^; N-low, 0.25 mM NO_3_^−^; N-complete, 7 mM NO_3_^−^) and were not (**A**) or were co-cultivated with *M. hyalina* (**B**). Amino acid profiles were identified 10 d after treatment. Data are given as the percentage of full NO_3_^−^ (7 mM NO_3_^−^) medium; n = 5–6.

**Figure 6:**
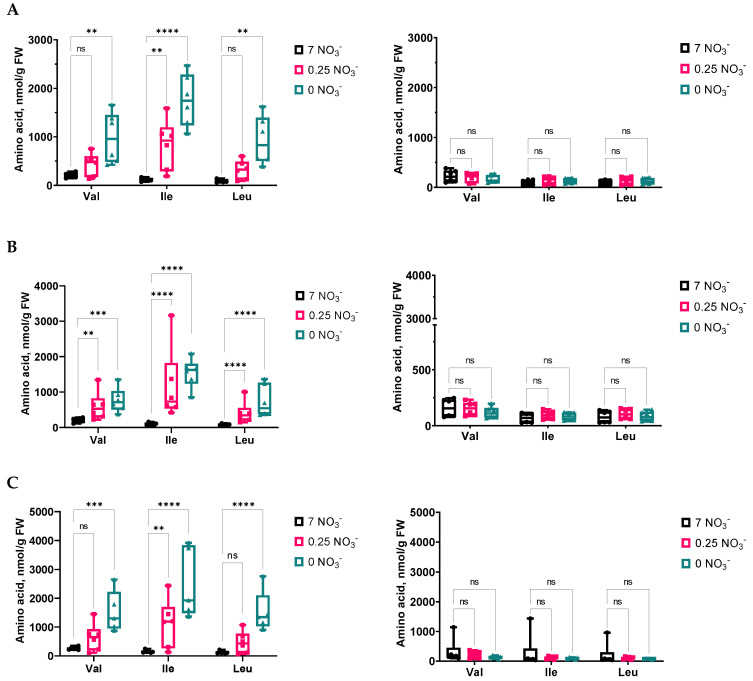
Branched-chain amino acids (BCAA) in shoots of *A. thaliana* Col-0 (**A**) and the ko mutants *nrt2.4-1* (**B**) and *nrt 2.4-2* (**C**) co-cultivated without (left) or with (right) *Mortierella hyalina* during NO_3_^−^ starvation. Two-week-old seedlings pre-grown on full NO_3_^−^ (7 mM NO_3_^−^) medium were further incubated on different NO_3_^−^ media (N-free, 0 mM NO_3_^−^; N-low, 0.25 mM NO_3_^−^; N-complete, 7 mM NO_3_^−^) and co-cultivated without (control) or with *M. hyalina.* Amino acids were measured 10 d after treatment. Two-way ANOVA with Dunnett’s multiple comparison test (data were transformed when needed); n = 5–6; ** *p* < 0.01; *** *p* < 0.001; **** *p* < 0.0001; ns: not significant.

Since the first organic compounds that carry absorbed N are amino acids, compositions and changes in the amino acid pools in the different Arabidopsis lines were analyzed individually in both the shoots and roots, depending on the given nitrate level in the medium, representing N starvation, and the presence/absence of *M. hyalina* (Figure 5). Looking deeper into the amino acid results, it is interesting to note that in all Arabidopsis lines, nitrate depletion had a particularly strong effect on the accumulation of branched-chain aliphatic proteinogenic amino acids (BCAA) such as leucine (Leu), isoleucine (Ile) and valine (Val) in the shoots (Figure 5A and Figure 6). All three amino acids were significantly accumulated at higher concentrations correlating with increasing nitrate deficiency. The presence of *M. hyalina* completely abolished this effect (Figure 5B and Figure 6).

**Figure 7:**
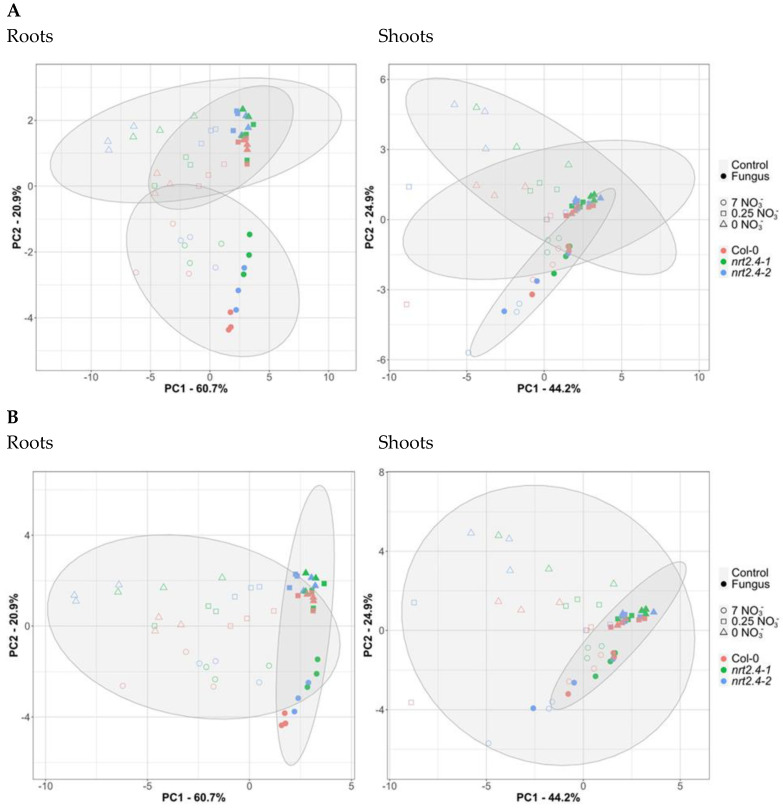
Principal component analyses (PCA) of amino acid compositions in *Arabidopsis thaliana* roots and shoots without or with *Mortierella hyalina* colonization. (**A**) The PCA score plot distinguishes the amino acid profiles of plants grown under different treatments of nitrate starvation (7 mM, 0.25 mM, no nitrate). (**B**) The PCA score plot distinguishes the amino acid profiles of plants grown without or with *M. hyalina* colonization. Two-week-old seedlings pre-grown on full NO_3_^−^ (7 mM NO_3_^−^) medium were further incubated on different NO_3_^−^ media (N-free, 0 mM NO_3_^−^; N-low, 0.25 mM NO_3_^−^; or N-complete, 7 mM NO_3_^−^) and were or were not co-cultivated with *M. hyalina*. Amino acid profiles were separately analyzed after 10 d. Open symbols, non-inoculated; closed symbols, *M. hyalina*-inoculated. The ellipses represent the multivariate normal distribution.

Moreover, a principle component analysis (PCA) of the amino acid composition in the roots revealed clear separation between the full medium (7 mM nitrate) on the one hand and the media with low and no nitrate (0.25 mM and 0 mM), respectively, on the other hand (Figure 7A). Here, the confidence areas (95%) of the data for no and low nitrate overlap almost completely. A similar clustering as in the roots was found for the shoots. The PCA of the amino acid composition in Arabidopsis roots and shoots that were and were not colonized by *M. hyalina* showed a cluster representing colonized plants, which is almost a sub-cluster of the non-colonized plants but clearly distinguishable (Figure 7B). This is even more obvious in the shoots. Interestingly, in the shoots, the sub-cluster of colonized plants also contains the non-colonized plants growing on 7 mM nitrate. This indicates that at least in the shoots, the fungus supports the plants so that their amino acid level is similar to if they were growing on the full medium. Strikingly, the Arabidopsis lines (Col-0, *nrt2.4-1*, *nrt2.4-2*) had no obvious impact. The two principal components, PC1 and PC2, explain in the roots 81.6% and in the shoots 69.1% of all observed variances.

## 3. Discussion

Fungal endophytes are an important component of the rhizosphere’s microbial communities. Many of these fungi are defined as commensalistic, with no or yet unknown functions in plants. However, some fungi have been shown to have negative (pathogens) or positive (mutualists) effects on their host plants. Typically, *Mortierella* species have a saprophytic lifestyle but they are also able to interact with and colonize many different plant species [23]. Similar to mycorrhizal fungi, some *Mortierella* spp. are supposed to support phosphate uptake into the plants, which may stimulate the biomass production of the host [29]. *M. hyalina* has been described as a beneficial fungus, promoting the growth of aerial plant tissues at least in the non-mycorrhizal plant *Arabidopsis thaliana* [24,25]. In addition, *M. hyalina* conferred tolerance against *Alternaria brassicae* infection [24]. However, whether or not and how *M. hyalina*, as with other beneficial fungi, can also rescue plants from abiotic stress such as nutrient deficiencies have not been studied so far. Thus, the effect of *M. hyalina* colonization on Arabidopsis plants with and without one component of the HATS for nitrate (NRT2.4 vs. *nrt2.4*) facing N starvation stress was investigated.

### 3.1. Growth Analysis of Arabidopsis Plants with/without Mortierella hyalina Colonization under Nitrogen Starvation

Growth analysis of Col-0 and the two *NRT2.4* ko lines showed no phenotypic abnormalities in the mutants at an optimal NO_3_^−^ supply, but did at low and no NO_3_^−^ concentrations (Figure 1A). According to the published data, no such difference in shoot growth has previously been found in the *nrt2.4-1* and *nrt2.4-2* lines [28]. However, the growth conditions in that study were different; actually, plants grew for 32 d on 0.5 mM NO_3_^−^ in short d and without added sugar. Interestingly, the strong dependency on nitrate concentration was not detected in the roots (Figure 1B), suggesting that the loss of function of NRT2.4 could be compensated for by other nitrate uptake systems. Indeed, *NRT2.5* was strongly induced in the nitrate-depleted plants, even with a different kinetics. While under low and no nitrate conditions, the *NRT2.4* gene was transiently but already highly expressed after 2 d, and *NRT2.5* was induced as well but with a different kinetics, i.e., much higher after 10 d than after 2 d of nitrate deficiency (Figure S2). This can explain why the NRT2.4 ko mutant plants can survive even with strong N deficiency (Figure 1) and show similar growth compared with the wild type. Nevertheless, the presence of *M. hyalina* has an additional impact on the plants’ growth. As shown in Figure 2, a promoting shoot over root growth effect was detectable in all plant lines, most pronounced in the wild type Col-0. Why this effect was neutralized under N deficiency is still not clear. It is conceivable that the fungus competes with the plant for the limited nitrogen and cannot promote plant growth anymore. However, even under such stress conditions, the fungus did not change its non-pathogenic nature and, thus, did not harm the plant.

### 3.2. Mortierella hyalina Mitigates the Arabidopsis Defense Responses

In any fungal plant colonization event, there is some potential for fungal virulence to facilitate infection, while host plant defenses can limit the development of fungal diseases [30]. A successful endophyte–host interaction involves a balance of the protagonists, regardless of the infected plant organ. In order to keep this balance and to establish a mutualistic relationship, the plant should not attack beneficial endophytic fungi during colonization. Thus, suppression of root immunity forms an important and very likely necessary background in the formation of plant-associated microorganisms’ communities [31]. In the interaction of *M. hyalina* with Arabidopsis roots, former studies described an increase of jasmonates after 1 d of co-cultivation, indicating that the plants initiated defense responses [32]. This increase appeared to be restricted to the early phase of interaction. Such a jasmonate accumulation was not detected in the present experiments, where jasmonates were measured after 10 days of colonization (Figure 3), confirming the former results in *M. hyalina*-colonized plants at later time points [24]. Moreover, even the N starvation-induced increase in jasmonates in the different Arabidopsis lines was reduced by the presence of the fungus (Figure 3). No *M. hyalina*-induced SA increase or even impact on the SA level was observed, neither in the former nor in this study. These results suggest that the fungus is able to mitigate the defense response and/or is accepted as a symbiotic partner by the host plant. Overall, fungal colonization initiated only a very weak defense response, while the abiotic N starvation stress increased the stress-related phytohormone levels, which the presence of *M. hyalina* attenuated mostly. This strengthens the view of a beneficial interaction between this fungus and the host plant.

### 3.3. Mortierella hyalina Does Not Modulate the NRT2.4 Induction in Arabidopsis under Nitrogen Starvation

The working hypothesis was that the presence of the fungus might support the plant in taking up NO_3_^−^ from the N-deficient media and, as a consequence, the well-known N-starvation-induced expression of *NRT2.4* [6,12] was not necessary. However, our data did not support this hypothesis, as shown in Figure 4. In the presence or absence of *M. hyalina*, the GFP under the control of the *NRT2.4* promotor was expressed under N deficiency at almost the same level with similar kinetics (Figure 4A). Nevertheless, the fungus clearly supplied the plants under N starvation with nitrogen; the less N was available in the medium, the more N was provided from the fungal stores (Table 1). Obviously, the N starvation sensory system of the plant did not recognize the fungal N supply very well, which might be due to the chemistry of the N, that is, whether it is inorganic or in a bound organic form, such as in amino acids.

### 3.4. Mortierella hyalina Restored the Amino Acid Homeostasis in Arabidopsis under Nitrogen Starvation

After uptake, nitrate is reduced into nitrite and ammonium (NO_3_^−^ → NO_2_^−^ → NH_4_^+^), while NH_4_^+^ is further incorporated into the amino acid glutamate, forming glutamine, the first organic compound that carries the nitrate-derived nitrogen. The enzyme glutamine synthetase mediates this reaction. Subsequently, many different aminotransferases distribute the amino group within the various amino acids and later into the whole plant metabolism. Thus, it was interesting to analyze the amino acid profile in the plants with and without fungal colonization as also postulated for interactions with beneficial bacteria [33]. The results obtained show clearly the N starvation effect on the Arabidopsis plants. Compared to the amino acid composition in plants growing without NO_3_^−^ stress, Col-0 as well as the ko mutant lines showed strong inconsistent changes in the amino acid levels, some of which accumulate to much higher levels (Val, Ile, Leu, His, Tyr, Trp, Lys), while others were strongly reduced (Arg, Asn, Gln) (Figure 5A). These effects were much more pronounced in the shoots compared with the roots. A corresponding PCA analysis that distinguished the amino acid profiles of plants grown under different levels of nitrate starvation (7 mM, 0.25 mM, no nitrate) indicated that in both the roots and shoots, all plants grown with full nitrate cluster together, as well as plants from low and no nitrate media (Figure 7A). The genetic background (WT, *nrt2.4-1*, *nrt2.4-2*) was less important than the nitrate concentration. Strikingly, when distinguishing between the amino acid profiles of plants grown with/without *M. hyalina* colonization, it became clear that in the roots, the colonized plants cluster together. In the shoots, colonized plants cluster together with most non-colonized Arabidopsis plants grown on a full nitrate medium, again independent of the genotype (Figure 7B). Based on these results, one can conclude that *M. hyalina* manipulates Arabidopsis so that even plants grown under N starvation gained an amino acid profile comparable with unstressed plants. The fungus restored the plants’ disturbed amino acid homeostasis to normal. It is still not known how this works mechanistically, whether or not the fungus provides selectively certain amino acids or has an impact on protein degradation and amino acid synthesis. These questions need to be addressed in further studies.

It is interesting to note that low nitrate stress had a clear effect on the accumulation of BCAA such as Leu, Ile and Val in shoots (Figure 5A and Figure 6). This finding has been mentioned before for Arabidopsis seedlings by Huang and Jander (2017) [34], who also described this phenomenon in response to drought, salt and osmotic stress, as well as herbicide treatment. In Arabidopsis, BCAA accumulation is primarily the result of protein degradation [34]. However, because neither ABA (Figure 3) nor proline levels (Figure 5) changed significantly upon N starvation, an osmotic stress response that also can induce BCAA accumulation must be excluded. The breakdown of amino acids produces intermediates or precursors of the tricarboxylic acid cycle (Acetyl-CoA) and thus contributes to the production of substrates for mitochondrial respiration. The oxidation of BCAA provides an amount of energy for ATP synthesis that is comparable to that provided by glucose [35]. The same holds true for lysine, which is also enriched under N starvation. Obvious is the tissue specificity for BCAA and lysine accumulation in the shoots rather than in the roots in all Arabidopsis lines and that the presence of *M. hyalina* largely eliminated the N starvation effect (Figure 5). However, to find out whether the N-starvation-induced increases in BCAA have a physiological function or are merely an artefact of protein degradation, more research needs to be pursued. In any case, upon protein degradation, the released amino acids are subsequently recycled and allocated for the biosynthesis of proteins required under nutrient limitation. The exact sensing of amino acid levels seems to be a key point for any efficient regulation of protein and amino acid metabolism. Thus, the regulation of amino acid content, flux and transport within the plant are critical for plant adaptation to nutrient status, as well as for development and stress responses.

As long as the molecular mechanisms underlying the coordination between plant growth and N metabolism are still not fully understood, significant improvement in controlled use of beneficial fungi is limited.

## 4. Materials and Methods

### 4.1. Plant Materials and Growth Condition

Different lines of *Arabidopsis thaliana* seeds were used: wild-type (ecotype Columbia-0) and transgenic line carrying the reporter construct *ProNRT2.4:GFP* [12]. Line *nrt2.4-1* was derived from a T-DNA–mutagenized population of the Col-0 accession [12,36], and *nrt2.4-2* (the SAIL line CS872100) was also derived from a T-DNA mutagenized population of the Col-0 accession [12,37].

*A. thaliana* seeds were surface-sterilized using 25% (*v*/*v*) sodium hypochloride (Acros Organics™, Bremen, Germany) and 0.1% of Triton X-100 (Sigma-Aldrich, Taufkirchen, Germany) for 8 min, rinsed seven times with sterile water and grown on square plates (120 × 120 × 16 mm) (Thermo Fisher Scientific, Dreieich, Germany) (12–15 seedlings per plate) containing MGRL medium (Table S1). The seeds were stratified for 48 h at 4 °C. The plants were incubated for 14 d in a growth chamber in vertical position under long-day conditions (16 h light/8 h dark) and a light intensity of 100 μmol photons m^−2^ s^−1^, at 22 °C.

For the different NO_3_^−^ concentrations, the *A. thaliana* seedlings (6 per plate) were transferred for 10 d onto MGRL N-free (0 mM NO_3_^−^), N-low (0.25 mM NO_3_^−^) and N-complete (7 mM NO_3_^−^) media, (1% sucrose, 0.5% Gelrite, pH 5.8) supplemented with KCl and CaCl_2_·2H_2_O in an appropriate quantity to support ion balance (Table S1).

These seedlings were further used for the different experiments without/with *M. hyalina* colonization. The control/fungal plugs (0.5 cm) were placed at a 0.5 cm distance from the plant root tips (Figure S1). Plants were harvested in threes in each vial (roots and shoots separately) and weighed. At least 18 seedlings from each treatment were taken. The samples were frozen immediately in liquid N, and stored at −80 °C for RNA preparation, and amino acid and phytohormone analysis. Only uniformly grown seedlings were used.

### 4.2. Phenotypic Analysis of Arabidopsis

Different lines of *A. thaliana* plants, after examining their growth phenotype on a NO_3_^−^-complete medium, were photographed on days 6 and 10 using a Samsung Galaxy A52 (Samsung Electronics Co., Seoul, Republic of Korea). The images were processed using Adobe Photoshop CS. The seedling root length (main and laterals) was measured using the Fiji ImageJ-2.9.0 Analysis software.

### 4.3. Mortierella hyalina Cultivation

The *M. hyalina* (FSU-509) strains were obtained from the Jena Microbial Resource Collection (Jena, Germany). The *M. hyalina* was cultured and maintained on Potato Dextrose Agar (PDA) medium (Sigma-Aldrich, Taufkirchen, Germany), at a pH of 5.6. Fungal plugs were transferred to the center of the PDA plates and incubated at 22–24 °C in the dark for 3 weeks in a growth chamber. as described by Johnson et al. (2019) [24].

To analyze whether *M. hyalina* can directly transfer N to the plant, it was labeled with ^15^N before co-culture with Arabidopsis. A modified KM medium without N-containing components (20 g/L dextrose, 50 mL/L macronutrients, 10 mL/L micronutrients and 1 mL/L Fe-EDTA, 1 mL/L vitamin mix, pH 6.5) was prepared and supplemented with 10 g/L ISOGRO^®^-^15^N (CortecNet, Les Ulis, France) according to the manufacturers’ protocol. *M. hyalina* plugs of 2 mm diameter were incubated (23 °C, 50 rpm, dark) in 2 mL of KM^ISOGRO^ for 10 days in Greiner CELLSTAR^®®^ 12-well plates (Greiner Bio-One, Frickenhausen, Germany) sealed with 3M^TM^ Micropore tape.

### 4.4. RNA Preparation and Expression Analysis

The total RNA (2.5 µg) was extracted using TRIzol, according to the manufacturer’s method, followed by additional chloroform isolation and isopropanol precipitation steps, digested to prevent DNA contamination using the TURBO DNA-*free*^TM^ KIT (Life Technologies, Carlsbad, CA, USA) and cleaned using the *RNA Clean and Concentrator™* KIT (™ trademarks of Zymo Research Corporation, Irvine, CA, USA). The cDNA (20 µL) was synthesized using an Thermo Fisher Scientific RevertAid First Strand cDNA Synthesis Kit (Thermo Fisher Scientific, Dreieich, Germany), according to the manufacturers’ instructions. The qPCR analysis was performed using a Bio-Rad CFX96^TM^ Real-Time System (Bio-Rad Laboratories Inc., Hercules, CA, USA) using the appropriate pairs for *A. thaliana*-specific primers (Supplementary, Table S2). The reaction components per 20 µL were as follows: 6.5 µL H_2_O, 12.5 µL Brilliant II SYBR Green qPCR Master Mix (Agilent Technologies, Santa Clara, CA, USA), 1 µL 10 µM of each primer and 1 µL cDNA. The thermal cycling program was as follows: initial denaturation at 95 °C for 180 s, and 44 cycles at 95 °C for 30 s, 60 °C for 30 s and 72 °C for 30 s. *AtActin 2* (AT3G18780) was used as an internal reference gene. The relative quantification of the gene expression was evaluated using the delta–delta Ct method according to Pfaffl (2001) [38]. Three biological replicates and three technical replicates were performed for each analysis.

### 4.5. Extraction and Quantification of Amino Acids Using LC–MS/MS

The plant material was homogenized in a Geno/Grinder^®®^ 2010 (Spex Sample Prep, Stanmore, UK) equipped with aluminum racks. The racks were cooled in liquid nitrogen before being used to prevent the thawing of the plant material throughout the homogenization process. The amino acids were extracted twice with a total of 2 mL of methanol on ice. The supernatants were combined and dried using a Concentrator plus (Eppendorf, Hamburg, Germany) and re-suspended in 500 μL of methanol. The extract was diluted 1:10 (*v*/*v*) with water containing the ^13^C, ^15^N-labeled amino acid mix (Isotec, Miamisburg, OH, USA) as the internal standard. The amino acids in the diluted extracts were directly analyzed using LC–MS/MS as described in [39] using a QTRAP 6500 mass spectrometer (Sciex, Darmstadt, Germany) coupled to an Agilent 1260 series HPLC system.

### 4.6. Extraction and Quantification of Phytohormones Using LC–MS/MS

The extraction procedure and phytohormone determination was carried out according to Müller et al. (2022) [39]. The tissue was extracted and homogenized in 1.5 mL methanol containing 60 ng D4-SA (Santa Cruz Biotechnology, Santa Cruz, CA, USA), 60 ng D6-JA (HPC Standards GmbH, Borsdorf, Germany), 60 ng D6-ABA (Toronto Research Chemicals, Toronto, ON, Canada) and 12 ng D6-JA-Ile (HPC Standards GmbH, Borsdorf, Germany) as the internal standards. Phytohormone analysis was performed using LC–MS/MS on an Agilent 1260 series HPLC system (Agilent Technologies, Santa Clara, CA, USA) coupled to a tandem mass spectrometer QTRAP 6500 (SCIEX, Darmstadt, Germany).

### 4.7. Analysis of Gene Expression in GFP Reporter Lines

Fluorescence microscopy of the GFP (green fluorescent protein) signals was optimized for live cells and detected in the roots at 10 d after transfer on a N-depleted medium after 24, 48 h and further on every 48 h. For the visualization of the GFP, images were acquired using a Zeiss AXIO Zoom.V16 (ZEISS, Oberkochen, Germany) equipped with a 0.5× PlanApoZ Objective (ZEISS, Oberkochen, Germany), an HXP120 mercury vapor lamp and a filter set 38 HE (excitation filter BP 450–490 nm, FT 495 nm, emission filter BP 500–550 nm). The signal intensities after treatment were measured using the Fiji ImageJ-2.9.0 Analysis software. The images were converted into 8-bit format and processed using Fiji’s “Analyze Particles” plugin. The average fluorescence intensity was measured in the cells of the apical lateral roots. For the measurement, 10 randomly selected fluorescent points in the form of a square of four pixels for each plant were used. Confocal images were captured using a STEDYCON imaging system (Abberior Instruments, Göttingen, Germany) on the 2nd d of N starvation. Excitation was evoked using a 488 nm laser diode and the detection range was around 526 nm. The final pixel size was 100 nm.

### 4.8. ^15^N Labeling Experiment

Labeled with ^15^N, after 10 d of growth, the fungal tissue was separated from the remaining medium and carefully washed 3 times with a N-free liquid MGRL medium to remove the ^15^N bound to the hyphal surface. The fungus was cut into 0.5 cm plugs and transferred into the MGRL (N-free, N-low and N-complete) plates for co-cultivation. To minimize the ^15^N uptake by the plant from the dead fungal material due to the washing and handling, the fungal plugs were placed at a minimum of 1 cm distance from the roots. Under these conditions, contact between the two organisms required the growth of hyphae toward the roots. Co-cultivation was performed for 10 d.

### 4.9. Isotope Analysis

Homogenous dry leaf powder (2–3 mg) was weighted in a tin capsule. δ^15^N isotope analyses were conducted using an elemental analyzer (NA1110, CE Instruments, Milan, Italy) coupled to a Delta+XL isotope ratio mass spectrometer (Thermo Finnigan, Bremen, Germany) via a ConFlo III. The sample element amounts were scaled against an in-house standard “Ali-j3” (Acetanilide) with δ^15^N values of −1.51 ± 0.1‰ on the δ^15^N AIR-N2 scales. “caf-j3” A (caffeine) sample was analyzed as a quality control with values of −15.46 ± 0.1‰ on the δ^15^N AIR-N2 scales [33]. Linearity, blank and drift corrections were undertaken for each sequence according to Werner and Brand (2001) [40].

### 4.10. Statistical Analysis

All the experiments were performed in accordance with the relevant guidelines and regulations. Independent experiments were treated as a completely randomized design. Figures were plotted using *GraphPad* Prism software version 9.0. The datasets of amino acids and phytohormones analyses were subjected to analysis using RStudio version 1.1.463 with R version 3.4.4. (R Development CoreTeam, 2018). Statistically significant differences were calculated using one- and two-way analysis of variance, with Dunnett’s multiple comparison test and Tukey’s post hoc test and a mixed-effects model (REML) with Dunnett’s multiple comparison test, with *p* < 0.05 as the threshold for significance.

## 5. Conclusions

Beneficial fungi can often mitigate abiotic-stress-induced physiological responses in their host plants. However, the underlying molecular mechanisms are largely unknown. In the present study, the role of the beneficial fungus *Mortierella hyalina* on *Arabidopsis thaliana* plants exposed to N starvation stress was investigated. One focus was on the hypothesis that fungal infection could alleviate N starvation stress by affecting the inducible high-affinity nitrogen transporter NRT2.4. This hypothesis could not be confirmed. Nevertheless, the results obtained show that the fungus has a positive effect on the plants. On the one hand, it is not recognized as a pathogen and the plant does not have to invest in the appropriate defense mechanisms but can continue to manage the nitrogen deficiency. On the other hand, it becomes clear that the fungus influences the amino acid metabolism and can restore the amino acid homeostasis disturbed by N starvation stress. This exemplifies how a beneficial fungus can support a plant under stress conditions and benefit from the symbiosis itself in the longer term. It also shows the potential of such interactions and possible mechanisms of how beneficial fungi can alter the metabolism of their host plants to mitigate stress symptoms and keep the plants alive.

## Figures and Tables

**Table 1:** δ^15^N level in shoots of *Arabidopsis thaliana* Col-0 colonized by ^15^N-labeled or unlabeled *Mortierella hyalina* fungi and the impact of different NO_3_^−^ concentrations in the growth medium.

	Unlabeled Fungi		Labeled Fungi	
	Delta vs. Air-corr. 29/28	%N corr. Concentration	Delta vs. Air-corr. 29/28	%N corr. Concentration
7 NO_3_^−^	4.70 ± 0.51	6.14 ± 0.12	242.04 ± 50.57	5.64 ± 0.24
0.25 NO_3_^−^	1.37 ± 0.38	1.49 ± 0.03	1134.98 ±259.92	1.55 ± 0.03
0 NO_3_^−^	0.21 ± 1.37	1.52 ± 0.06	2077.79 ± 330.63	1.45 ± 0.05

Two-week-old seedlings pre-grown on full NO_3_^−^ (7 mM NO_3_^−^) medium were further incubated on different NO_3_^−^ media (N-free (0 mM NO_3_^−^), N-low (0.25 mM NO_3_^−^) and N-complete (7 mM NO_3_^−^)) and co-cultivated with *M. hyalina* for another 10 d. *M. hyalina* was pre-grown for 10 d either on ^15^N-labeled or unlabeled amino acids.

## Data Availability

The data supporting the findings of this study are available on request from the corresponding author.

## Supplementary Materials

The following supporting information can be downloaded at https://www.mdpi.com/article/10.3390/ijms242216128/s1.
