# Supplementary material for: The Beneficial Fungus Mortierella hyalina Modulates Amino Acid Homeostasis in Arabidopsis under Nitrogen Starvation
Source: Int J Mol Sci. 2023 Nov 9;24(22):16128. doi: 10.3390/ijms242216128 (PMC10671455; doi:10.3390/ijms242216128)
Supplement: Supplementary file 1 [file ijms-24-16128-s001.zip › ijms-2681131-supplementary.pdf]

Supplemental Table S1: MGRL medium composition.

Nutrients/Chemicals	MGRL/7mM NO_3^-	MGRL/0.25 mM NO_3^-	MGRL/0 mM NO_3^-
KNO_3	3 mM	0.11 mM	NA
$Ca(NO_3)_2{\cdot}5H_2O$	2 mM	0.07 mM	NA
KCl	NA	2.89 mM	3 mM
$CaCl_2{\cdot}2H_2O$	NA	1.93 mM	2 mM
$Na_2HPO_4{\cdot}7H_2O$ (pH=5.8)	85.3 μM	85.3 μM	85.3 μM
$NaH_2PO_4{\cdot}H_2O$ (pH=5.8)	1.67 mM	1.67 mM	1.67 mM
$MgSO_4{\cdot}7H_2O$	1.5 mM	1.5 mM	1.5 mM
Na_2-EDTA·$2H_2O$	67 μM	67 μM	67 μM
H_3BO_3	30 μM	30 μM	30 μM
$MnSO_4{\cdot}7H_2O$	10.3 μM	10.3 μM	10.3 μM
$FeSO_4{\cdot}7H_2O$	8.6 μM	8.6 μM	8.6 μM
$CuSO_4{\cdot}5H_2O$	1 μM	1 μM	1 μM
CoCl2·$6H_2O$	130 nM	130 nM	130 nM
$(NH_4)_6Mo_7O_{24}{\cdot}4H_2O$	24 nM	24 nM	24 nM
$ZnSO_4{\cdot}7H_2O$	1 μM	1 μM	1 μM
Sucrose	1.0% w/v	1.0% w/v	1.0% w/v
Gelrite™	0.5% w/v	0.5% w/v	0.5% w/v

Supplemental Table S2: Oligonucleotides used for quantitative RT-PCR.

Primer name	Sequence 5′→ 3′	Targets
AtNRT2.4 forward	CAGTTCCTTCCGACTCATCA	AT5G60770
AtNRT2.4 reverse	GCAACACCAGCATTTCCGAC	AT5G60770
AtNRT2.5 forward	CTCCTCCCTGTTATCCGTGAAA	AT1G12940
AtNRT2.5 reverse	AGACGAAAGTGGCGAGAGAGAA	AT1G12940
AtActin 2 forward	GGAATCCACGAGACAACCTA	AT3G18780
AtActin 2 reverse	ATCTTCATGCTGCTTGGTGC	AT3G18780

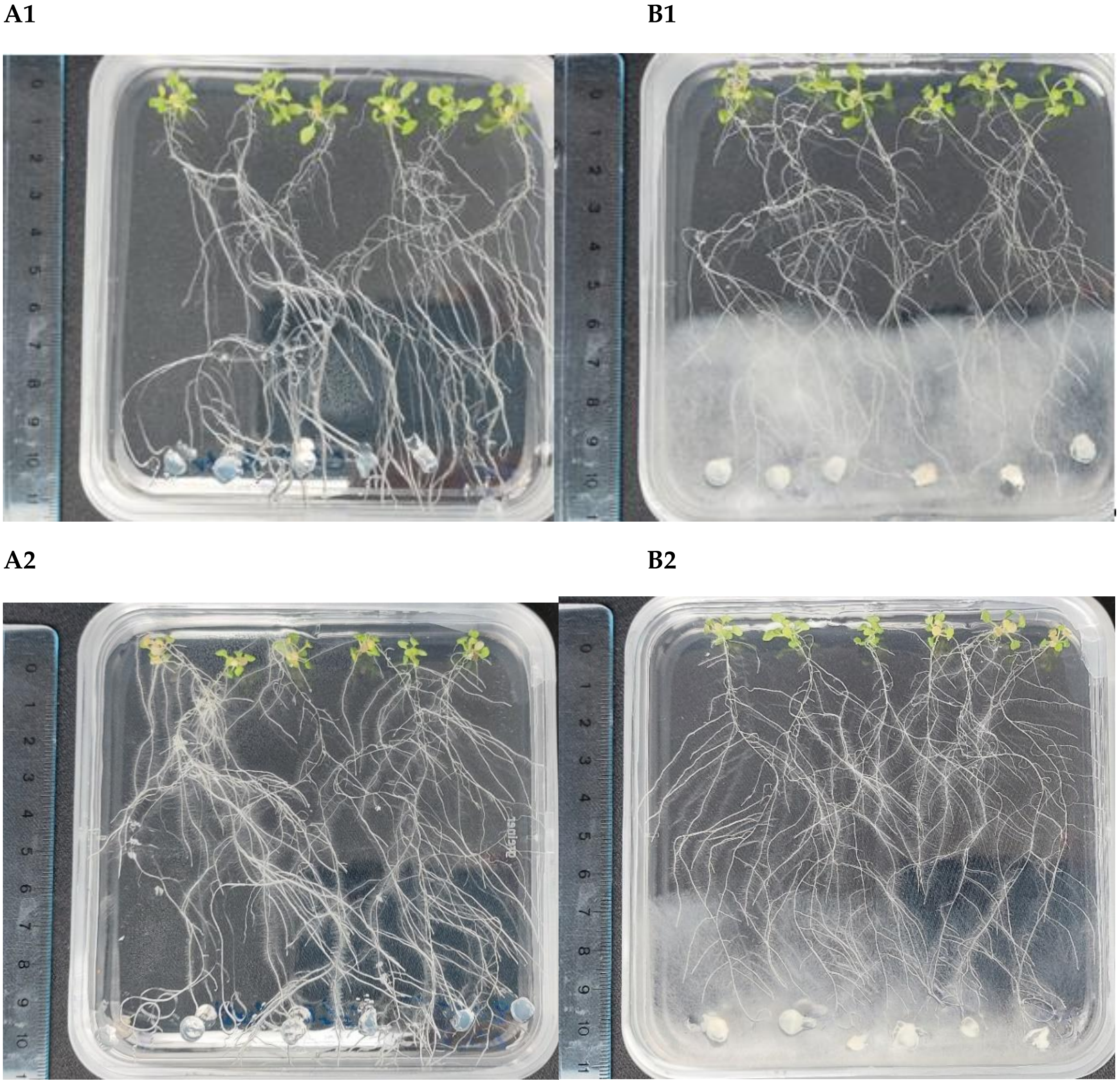

Supplemental Figure S1. Co-cultivation of *Arabidopsis thaliana* Col-0 plants with *Mortierella hyalina*: 20-day-old-plants (6 day after co-cultivation) growth on MGRL medium containing 7mM (A1, B1) or 0 mM (A2, B2) NO_3^-. Control plants (A), co-cultivated with *M. hyalina* (B); see control and fungal plugs at the bottom.

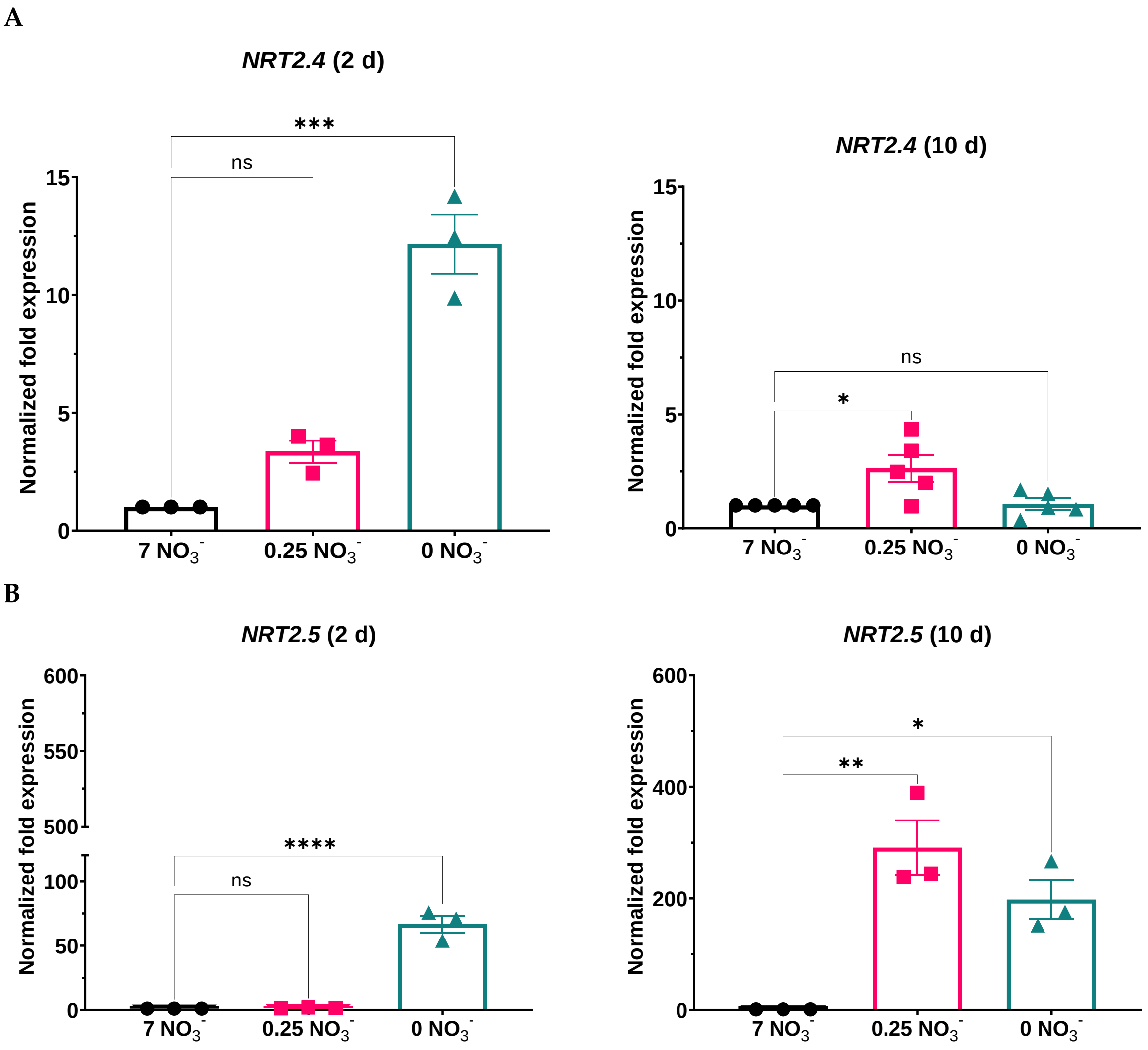

Supplemental Figure S2. *NRT2.4* (A) and *NRT2.5* (B) expression in roots of *Arabidopsis thaliana* WT. Two-weeks-old seedlings pre-grown on full NO_3^- (7 mM NO_3^-) medium were further incubated on different NO_3^- medium (N-free, 0 mM NO_3^-; N-low, 0.25 mM NO_3^-; or N-complete, 7 mM NO_3^-). One-way ANOVA with Dunnett's multiple comparisons test; the error bars indicate standard error of the mean (SEM); (n = 3–5); *P<0.05; **P<0.01; *** P<0.001; **** P<0.0001; ns: not significant.
